# Lessons from polio eradication: a synthesis of implementation strategies for global health services delivery from a scoping review

**DOI:** 10.3389/frhs.2024.1287554

**Published:** 2024-08-07

**Authors:** Abigail H. Neel, Adetoun Olateju, Michael A. Peters, Meike Schleiff, Olakunle Alonge

**Affiliations:** ^1^ Department of International Health, Johns Hopkins Bloomberg School of Public Health, Baltimore, MD, United States; ^2^ Sparkman Center for Global Health, University of Alabama at Birmingham, Birmingham, AL, United States

**Keywords:** implementation research, implementation strategies, implementation outcomes, polio, global polio eradication initiative

## Abstract

**Introduction:**

There is limited guidance on strategies for delivering complex global health programs. We synthesized available evidence on implementation strategies and outcomes utilized in the global polio eradication initiative (GPEI) across low and middle-income country (LMIC) settings.

**Methods:**

We nested our scoping review into a literature review conducted as part of a parent study, STRIPE. This review systematically searched PubMed for articles between 1 January 1988 and 25 April 2018 using polio search terms. Strategies from included studies were organized according to the Expert Recommendations for Implementing Change (ERIC) framework, specified using Proctor's framework, and linked to various outcomes (implementation, services delivery, impact).

**Results:**

152 unique articles fulfilled our inclusion criteria (from 1,885 articles included in the parent study). Only 43 out of the 152 articles described a suitable quantitative study design for evaluating outcomes. We extracted 66 outcomes from the 43 unique studies. Study publication dates ranged from 1989 to 2018 and represented diverse country settings. The most common implementation strategies were developing mechanisms for feedback, monitoring, and evaluation (*n* = 69); increasing awareness among the population (*n* = 58); involving stakeholders, workers, and consumers in the implementation efforts (*n* = 46); conducting workshops (*n* = 33); using mass media (*n* = 31); and building robust record systems to capture outcomes (*n* = 31). Coverage (*n* = 13) and morbidity (*n* = 12) were the most frequently identified outcomes, followed by effectiveness (*n* = 9) and fidelity (*n* = 6). Feasibility and sustainability were rarely evaluated.

**Conclusions:**

This review provides a catalogue of implementation strategies and outcomes relevant for advancing global health services delivery in LMICs drawing from the GPEI. Implementation strategies reviewed were poorly described and not adequately linked to outcomes. It calls for additional implementation research to unravel the mechanisms of implementation strategies and their effectiveness, and adaptation of the ERIC framework in LMICs.

## Background

It can be challenging for public health practitioners to identify implementation strategies that will be the most effective for achieving desired health outcomes, and to determine which strategies may be the most relevant given the characteristics of both the intervention and implementation context. This challenge is exacerbated by a lack of adequate, comparable descriptions of implementation strategies within implementation science literature, and of the contextual barriers and outcomes that these strategies address (1, 2). Many studies fail to elaborate who delivers the implementation strategy, how the strategy is deployed, i.e., the processes or steps involved, the target of the strategy, and the frequency and intensity required for the strategy to be effective (3). Without a clear understanding of these features, practitioners may struggle to appropriately select and evaluate implementation strategies for addressing barriers to and facilitators of change, prioritize empirical evidence on implementation strategies from other contexts, and learn from and adapt evidence-supported implementation strategies to their prioritized issue and context. Researchers and practitioners alike will struggle to translate findings from ongoing disease control efforts into real-world applications. This gap is especially important in low- and middle-income countries where resources may be lacking to conduct locally based large-scale effectiveness studies around implementation strategies, and where actors may benefit from drawing on and adapting evidence from other settings. A synthesis of available evidence on implementation strategies, which seeks to describe how, when, and to what effect implementation strategies may be used is therefore needed.

The Expert Recommendations for Implementing Change (ERIC) framework provides a taxonomy for classifying implementation strategies, covering domains including management and problem-solving, monitoring and evaluation, engagement and capacity building, and communications and advocacy (3). By systematically gathering input on implementation strategies, the ERIC taxonomy provides consensus definitions on implementation strategies relevant to health services delivery. Given the lack of clarity on implementation strategies in the literature, the ERIC framework provides a useful conceptual home for understanding the breadth of implementation strategies utilized in a global program. Still, as the authors acknowledge, because ERIC was developed by and for stakeholders in North America, and drawn largely from high-income country settings, its transferability across contexts and applicability to low and middle-income settings may be limited. In this paper we have leveraged the ERIC framework to systematically describe the implementation strategies deployed for implementing the global polio eradication initiative (GPEI) while reflecting on its fit for global programs.

The GPEI provides a rich landscape for this assessment for several reasons. First, both the longevity and intensity of the initiative have contributed to a proliferation of research focused on the implementation of eradication activities, including the enactment of different implementation strategies, and polio-related health outcomes. Second, as a truly global initiative and one of the largest of its kind (4), the GPEI adopted a global strategy which was applied, and necessarily adapted and refined, across diverse low- and middle-income countries (LMIC) and regional contexts. While the *programmatic* strategies for the GPEI (i.e., surveillance, routine immunization, supplementary immunization activities, and mop-up campaigns) are conceptually distinct from the *implementation* strategies utilized to enable them, the global nature of the initiative facilitated multi-country application of implementation strategies, both through the efforts of implementing partners [e.g., WHO, United Nations Children's Fund (UNICEF), Rotary International, the U.S. Centers for Disease Control (CDC), the Bill and Melinda Gates Foundation (BMGF), the CORE group], and through national ministries of health and frontline health workers working in concert with global guidelines, procedures, and tools (5, 6). Thus, the GPEI provides a useful opportunity for assessing and synthesizing empirical evidence on various implementation strategies across diverse contexts, and the factors which may have led to variation in the effectiveness of select strategies, to facilitate the translation of these implementation strategies to other programs and settings.

For our study, implementation strategies are defined as “methods or techniques used to enhance the adoption, implementation, and sustainability of a clinical program or practice” (7, 8) [though, as per Peters et al. (9), we define clinical program or practice to include population-based public health interventions as well as individual clinical interventions]. The goal of this study is to describe the implementation strategies used throughout the Global Polio Eradication Initiative, including the challenges selected strategies were aimed to address and how they were operationalized, and to reflect on the strengths and limitations of available evidence. In the results that follow, we have aimed to synthesize this evidence by categorizing and describing implementation strategies utilized throughout the initiative from 1988-present according to the ERIC framework, the different types of outcomes they influenced, and their impact in diverse LMIC settings.

## Methods

Our methodology followed the PRISMA Extension for Scoping Reviews (PRISMA-ScR) (as specified in Supplementary File S1) (10).

### Search strategy

We nested our scoping review into a broader literature review of the GPEI conducted as part of a parent study, the *Synthesis and Translation of Research and Innovations from Polio Eradication (STRIPE)* (6)*.* This review searched the electronic database PubMed for articles between 1 January 1988 (to align with the year the GPEI began) and 25 April 2018, using search terms for polio, and the search strategy and methods are described elsewhere (5). (Supplementary File S2 specifies the search terms used). Given the breadth of peer-reviewed literature on polio eradication implementation activities included in this initial review, we decided to pursue a secondary analysis focused on synthesizing implementation strategies utilized in the effort, and outcomes measured. We retained those articles included in the full text review of the scoping review (i.e., relevant to implementation of the GPEI in low and middle-income countries), and which were categorized as original/research articles or review articles (5). While the review was inclusive only up until 2018, we expect the review to still enclose sufficient data to generate a comprehensive synthesis given that the included 30 years period is when GPEI activities were at their peak.

### Inclusion and exclusion criteria

**Figure 1:**
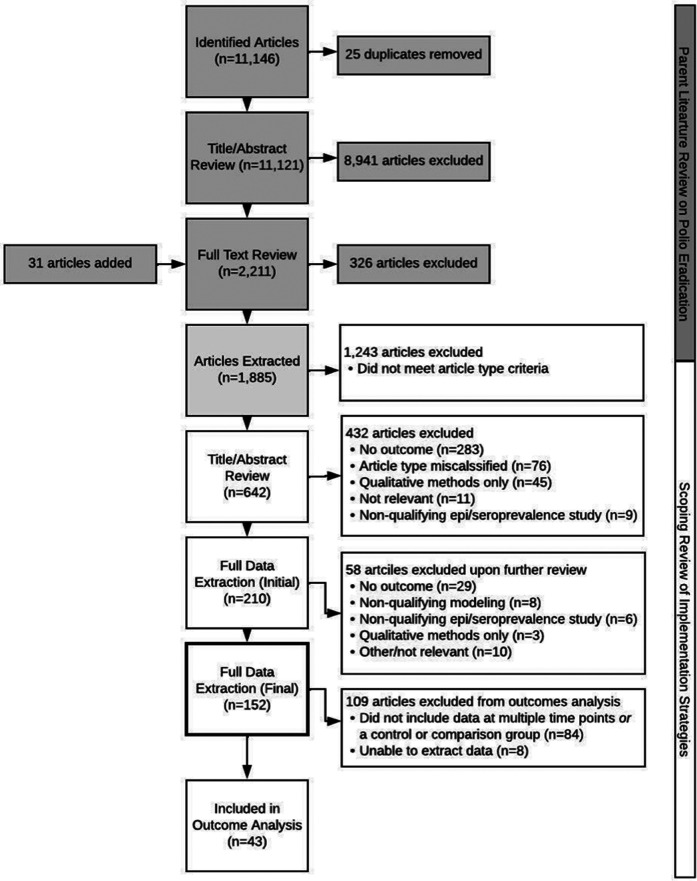
PRISMA flow diagram.

Since relevance to GPEI implementation was established *a priori* along with time, geography, and language restrictions (5), we focused on developing exclusion criteria to remove any articles not directly related to our aim of quantitatively assessing the effectiveness of implementation strategies utilized in polio eradication. These criteria are described in full in Supplementary File S3, but in short, articles were excluded if they (1) utilized only qualitative methods; (2) did not measure implementation, service delivery, or impact-level outcomes; (3) reported on epidemiological or seroprevalence studies that did not include at least two time points or a comparison district; (4) were modeling studies assessing non-programmatic features, or (5) did not meet article type criteria (e.g., an original/research or review article). Conversely, articles were included if they met inclusion criteria for the GPEI scoping review, were categorized as an original/research or review article, and included quantitative measurement of one or more implementation, service delivery, or impact-level outcomes. While valuable and included in the parent study, grey literature was excluded from this analysis as we sought to assess how published literature reported on and measured outcomes related to implementation. Two analysts independently reviewed titles and abstracts for inclusion in full text review. Conflicts were clarified at the midpoint, and final resolutions were completed at the endpoint by a third researcher. While we aimed to be comprehensive in our review, we may have missed some relevant data by excluding non-English language articles, as well as articles that were not deemed original/research or review articles. For the outcomes analysis only studies that included data collected at multiple timepoints and/or control or comparison groups (*n* = 43) were included (see Figure 1).

### Data extraction strategy

A data extraction tool was developed and used to collect information on study design, implementation strategies, and outcomes. The data extraction tool asked analysts to determine inclusion/exclusion of the material, identify implementation strategies applied in the study, identify the outcome type, and assess elements of the study design, including methods, data sources, study type (e.g., randomized control trial, stepped wedge, etc.), frequency of data collection, and whether control or comparison groups were used. Following a pilot test of six articles and subsequent revisions to the data extraction tool, four analysts were randomly assigned a batch of articles for full text review. Ten percent of articles included were reviewed by all four analysts. The overall percent agreement across all 392 variables in our extraction tool was 86% (Benchmark Interval: 80%–100%), implying almost perfect agreement among raters based on the benchmark scale without accounting for agreement due to chance. A full analysis of inter-rater reliability conducted in Stata (14.2) on selected variables is available in Supplementary File S4 (11).

Data was collected on three key areas: study design, implementation strategies, and outcomes. We assessed characteristics of the study design as they are related to the level of inference (12), including sample size, study methods, data sources, data collection timepoints, and use of control and comparison groups. Implementation strategies were extracted as part of the original GPEI scoping review according to an adapted ERIC taxonomy (13). We also assessed the socioecological level at which the strategy was deployed, e.g., individual, household, community, organizational, system-wide. Outcome types were defined according to pre-existing typologies (9, 14) and categorized as implementation, service delivery, or impact (morbidity, mortality) outcomes. Implementation outcomes were defined as “effects of deliberate and purposive actions to implement new treatments, practices, and services” and service delivery outcomes derived from the Institute of Medicine (IOM) (8). In addition, the value, statistical significance, and direction of effect measures were extracted where available. To assure the quality of the included articles, the outcome analysis examining the effect or impact of implementation strategies was restricted only to studies with a comparison group for the implementation strategy, and/or outcome data collected over at least two timepoints.

### Data synthesis and presentation

To standardize our results, we have drawn from a number of existing implementation science theories to describe the strategies in detail, including the ERIC framework which provides definitions for common implementation strategies (3), the Consolidated Framework on Implementation Research (CFIR) which provides a menu of constructs associated with effective implementation and contextual variables that may be the target of implementation strategies (15) what we have described here as “action targets”, as well as Proctor et al.'s guidance for specifying and reporting implementation strategies (8).

Proctor et al.'s guidance suggests that to fully describe implementation strategies they must be named, defined, and operationalized (8). We used the Expert Recommendations for Implementing Change (ERIC) catalog of implementation strategies (3) to name and define the implementation strategies used in included studies; this was part of the initial STRIPE study, whose methodology has been described more fully elsewhere (5, 6), but we used ERIC in order to have a standardized set of implementation strategies that would enable comparison across initiatives. We then described how these strategies were operationalized by describing the actor, action, dose, temporality and justification using 1–2 representative studies, selected to describe *how* and *why* implementation strategies were utilized within the polio eradication initiative. To standardize how we described the action targets of these strategies, we leveraged the CFIR framework, which also guided the STRIPE project and includes a series of domains within which implementation strategies may be levied (15). Finally, based on review of the included studies, we identified the implementation outcomes which were most often targeted by the implementation strategies deployed.

## Results

### Overview of selected studies

Figure 1 presents the PRISMA flow diagram of the study selection process (16), bifurcated to illustrate the initial GPEI scoping review conducted as part of the STRIPE project and the nested implementation strategies and outcomes analyses reported in this paper. We retained 642 articles for title-abstract review after removing articles that were included in the scoping review but did not meet our requirements for article type (*n* = 1,243), i.e., were not an original/research or review article. A total of 432 articles were subsequently excluded during the abstract screening, most commonly because the article did not include an effectiveness measure of implementation strategies deployed for polio eradication (51.79%). Two-hundred and ten articles were identified for data extraction (see Supplementary File S5 for an overview of these 210 articles). An additional 58 articles were excluded from full data extraction upon further review: 29 did not have an outcome, 8 were modeling studies that did not model relevant outcomes, 6 were seroprevalence studies without an implementation strategy, 3 employed qualitative methods only, and 12 were excluded for a variety of other reasons. Ultimately, 152 articles were included in full text extraction (Tables 1–5). A subset of these articles (*n* = 68) included data at multiple timepoints (*n* = 51) and/or utilized control or comparison groups in their measurement of implementation, service delivery, or impact outcomes (*n* = 17), and were thus assessed to be of higher quality, were included for consideration in the outcome analysis (Tables 6–8). An additional 8 articles were excluded from inclusion in Tables 6–8 upon analysis as analysts were unable to extract data or access the full text articles.

**Table 1:** Characteristics of included articles (*n* = 152).

	# (%) of articles
Region	
African Region (AFR)	69 (45.39)
Eastern Mediterranean Region (EMR)	24 (15.79)
European Region (EUR)	12 (7.89)
Region of the Americas (AMR)	43 (28.29)
South-East Asia Region (SEAR)	14 (9.21)
Western Pacific Region (WPR)	0 (0)
Global	8 (5.26)
Publication timeframe	
1988–2000	26 (17.01)
2001–2012	46 (30.27)
2013–2018	80 (52.63
Study design	
Case control	6 (3.95)
Cohort	2 (1.32)
Cross-sectional	81 (53.29)
Dose response	1 (0.66)
Modeling	41 (26.97)
Other	6 (3.95)
Control and comparison group use	
Equivalent	1 (0.66)
Non-equivalent	16 (10.53)
No control/comparison	134 (88.16)
Missing	1 (0.66)
Included data at multiple time points	
Yes	51 (33.55)
No	101 (66.45)

**Table 2:** Implementation strategies: management & problem solving^a^.

Implementation strategy	Definition	How operationalized *(actor, action, dose, temporality)*	Action target *(CFIR)*	Implementation outcomes affected	Justification
Assess organizational ability and readiness *(n = 18)*	Assess various aspects of an organization to determine its degree of readiness to implement, barriers that may impede implementation, and strengths that can be used in the implementation effort	• Conducting an evaluation to assess the cold chain's adaptability to inactivated poliovirus vaccine (IPV) introduction (Bangladesh) (17) • Identifying bottlenecks to service delivery in high-risk/low coverage districts (Pakistan) (18)	Inner setting	Fidelity, coverage	Allow adaptation based on emerging barriers
Adapt physical structures and equipment to interventions *(n = 8)*	Evaluate current configurations and adapt, as needed, the physical structure and/or equipment (e.g., changing the layout of a room, adding equipment) to best accommodate the targeted innovation	• Setting-up temporary “health camps” to deliver non-polio, ambulatory services as well as polio vaccination (Nigeria) (19, 20) • Setting-up immunization campaigns to deliver vaccination in hard-to-reach areas (Ethiopia) (21)	Intervention characteristics	Acceptability, coverage	Improve acceptability of polio services; increase access in conflict and hard-to-reach areas
Build robust record systems to capture outcomes *(n = 31)*	Develop record systems to allow better assessment of implementation or clinical outcomes	• Using the clustered lot quality assurance (c-LQAS) sampling technique to identify low coverage districts post immunization campaign (Cameroon) (22) • Developing an accountability framework to track key program performance indicators (Ethiopia) (23)	Inner setting, process	Fidelity, coverage	Guide mop-up activities to address pockets of low coverage; improvements in staff and program performance
Model and simulate desired changes and outcomes *(n = 16)*	Model or simulate the change that will be implemented prior to implementation	• Conducting economic analyses to estimate program cost, cost-benefit ratios, and economic costs saved (global) (24) Developing novel methods for assessing and predicting campaign effectiveness (Nigeria) (25)	Inner setting, process	Coverage, cost	Justify GPEI program investment; allow for comparison of multiple immunization calendars
Identify or build a dissemination organization *(n = 10)*	Identify or start a separate organization that is responsible for disseminating the clinical innovation. It could be a for-profit or non-profit organization	• Engaging existing youth groups before, during, and after immunization campaigns, training them to accompany vaccine teams (Nigeria) (26) Forming dedicated mobile vaccination and community mobilization teams to support implementation in high-risk districts (Nigeria) (27)	Outer setting	Acceptability, Coverage	Reduce vaccination team harassment and improve community compliance; improve coverage in high-risk districts
Centralize assistance for implementation issues*(n = 8)*	Develop and use a centralized system to deliver technical assistance focused on implementation issues	• Creating a committee on poliomyelitis to study the problems related to polio prevention and inform national strategy (Singapore) (28) • Utilizing initiative-led process for accrediting laboratories (AFRO region) (29)	Inner Setting	Coverage, Fidelity	Create shared understanding of implementation barriers; standardize quality across sites
Offer incentives or disincentives to providers and consumers*(n = 15)*	Provide financial disincentives for failure to implement or use the clinical innovations	• Offering diagnostic and prescription services as an incentive to attend polio-supported “health camps” (Nigeria) (19) • Offering preventive services, e.g., oral rehydration solution (ORS) and insecticide-treated nets (ITNs) distribution, Tetanus Toxoid vaccination, malnutrition, and HIV screenings as part of Supplementary Immunization Activities (SIAs) (Nigeria) (30)	Outer setting	Coverage, fidelity, acceptability	Generate participation in service delivery campaigns

^a^ In Tables 2–5, the number of articles that reported on a given implementation strategy (column one), the “action target” (column four) and “implementation outcomes affected” (column five) reflect analysis results from all included studies. Other domains, e.g., “how operationalized” (column three) and “justification” (column six) reflect results from 1 to 2 representative studies, selected to describe how and why implementation strategies were utilized within the polio eradication initiative.

**Table 3:** Implementation strategies: monitoring & evaluation^a^.

Implementation strategy	Definition	How operationalized *(actor, action, dose, temporality)*	Action target *(CFIR)*	Implementation outcomes affected	Justification
Develop mechanisms for feedback, monitoring, and evaluation *(n = 69)*	Develop and organize systems and procedures that monitor implementation processes and/or outcomes for the purpose of quality assurance and improvement	• Conducting post-campaign monitoring using mobile technology to contact recipients (Pakistan) (31) • Deploying designated monitors across countries to validate the trivalent to bivalent oral poliovirus vaccine (tOPV, bOPV) switch (global) (32) • Developing household-based microplanning (Nigeria) (33)	Process	Coverage, fidelity	Verify program implementation (e.g., coverage of SIAs, removal of tOPV); improve population enumeration and identify for follow-up chronically missed children
Conduct cyclical small tests of change *(n = 1)*	Implement changes in a cyclical fashion using small tests of change before taking changes system-wide. Tests of change benefit from systematic measurement, and results of the tests of change are studied for insights on how to do better. This process continues serially over time, and refinement is added with each cycle	• Conducting rapid process evaluation at the beginning of implementation, and course correcting for subsequent pulse polio immunization days (India) (34)	Intervention characteristics, process	Fidelity	Allows for rapid retooling of implementation processes to ensure fidelity
Create credentialing and liability standards *(n = 1)*	Create an organization that certifies clinicians in the innovation or encourage an existing organization to do so. Change governmental professional certification or licensure requirements to include delivering the innovation. Work to alter continuing education requirements to shape professional practice toward the innovation	• Utilizizing initiative-led process for accrediting laboratories (AFRO Region) (29)	Inner setting, characteristics of individuals	Fidelity	Accreditation process demonstrates capacity to detect, identify, and report WPV and annual reviews ensure fidelity to WHO laboratory procedures
Visit other sites where similar efforts have been successful *(n = 2)*	Visit sites where a similar implementation effort has been considered successful	• Documenting best practices across settings in Africa (AFRO Region) (35) • Participating in cross-country learning trips. Found that increasing technical staff at sub-national levels accelerated polio eradication and adopted the “Indian technical surge capacity model” (India, Nigeria) (36)	Inner setting, outer setting	Penetration efficiency	Accelerated and sustained the implementation of quality supplemental immunization activities at the LGA, ward, and settlement levels in 11 high-risk priority states

^a^ In Tables 2–5, the number of articles that reported on a given implementation strategy (column 1), the “action target” (column 4) and “implementation outcomes affected” (column five) reflect analysis results from all included studies. Other domains, e.g., “how operationalized” (column three) and “justification” (column six) reflect results from 1 to 2 representative studies, selected to describe how and why implementation strategies were utilized within the polio eradication initiative.

**Table 4:** Implementation strategies: engagement & capacity building^a^.

Implementation strategy	Definition	How operationalized *(actor, action, dose, temporality)*	Action target *(CFIR)*	Implementation outcomes affected	Justification
Build multidisciplinary partnerships and coalitions *(n = 14)*	Recruit and cultivate relationships with partners in the implementation effort	• Engaging trusted sources of information including teachers, community, and religious leaders to participate in health communication and OPV administration (Nigeria) (37) • Holding community meetings (e.g., with nomadic community leaders, veterinary service staff, local government administrators) to identify nomadic population movement (Chad) (38)	Outer setting, process	Coverage, penetration	Increase demand and uptake in low-performing districts; help locate hard-to-reach populations
Leverage existing collaborations and networks *(n = 22)*	Facilitate the formation of groups of providers and organizations and foster a collaborative learning environment to promote information sharing, collaborative problem solving, and a shared goal to improve implementation.	• Engaging youth groups to accompany vaccination teams in hostile communities (Nigeria) (26) • Collaborating with transport stakeholders to vaccinate mobile populations in transit (SEARO Region) (39) • Providing services for livestock and children to immunize nomadic populations (Somalia) (40)	Inner setting, outer setting, process	Coverage, fidelity (Penetration, acceptability)	Engage relevant stakeholders and networks to improve coverage and fidelity of vaccination programs
Involve stakeholders, workers, and consumers in the implementation efforts *(n = 46)*	Involve existing governing structures, engage consumers and communities in the implementation effort	• Developing a social mobilization network (SM Net) of partners to develop behavior change communication materials, standardize field staff positions, and engage community mobilization coordinators and *Bullawa tollies* (India) (41) • Conducting community engagement meetings with local leaders prior to outreach activities (Nigeria) (42)	Outer setting, process	Coverage, acceptability	Increase vaccine uptake and acceptability of activities in target districts; verify settlement information with local leaders to ensure coverage
Capture and share knowledge, opinions, and needs *(n = 27)*	Capture local knowledge from implementation sites on how implementers and clinicians made something work in their setting and then share it with other sites	• Surveying perceptions and knowledge of health workers involved in supplemental immunization activities (Nigeria) (43) • Understanding community perceptions of OPV and reasons for vaccine refusal (Pakistan) (44)	Process	Acceptability, coverage	Engage with supply- and demand-side actors to understand perceptions of aspects of the project and overcome bottlenecks to program delivery
Make training dynamic and varied *(n = 9)*	Vary the information delivery methods to cater to different learning styles and work contexts, and shape the training in the innovation to be interactive	• Monitoring and accountability officers follow-up on action plans after didactic training (Nigeria) (45) • Conducting training of microplan supervisors and enumerators on revised microplanning concepts as part of preparatory stage of 6-part microplanning process (Nigeria) (33)	Inner setting, characteristics of individuals, process	Coverage, fidelity	Prepare implementers with correct tools and ensure training aims are realized in the field
Recruit, designate, and train leaders *(n = 12)*	Recruit, designate, and train leaders for the change effort	• Facilitating intersectoral collaboration (e.g., with Federal Road Safety Corps, National Union of Road Transport Workers, and market leaders) in transit polio vaccination campaigns (Nigeria) (46) • Training and deployment of polio volunteer community mobilizers and dedicated mobile teams (Nigeria) (27)	Inner setting, characteristics of individuals, process	Coverage, acceptability	Attract communities to become involved in eradication activities to increase program reach
Use train-the-trainer strategies *(n = 2)*	Train designated clinicians or organizations to train others in the clinical innovation	• Conducting cascade training among surge capacity personnel on relevant expanded programme on immunization (EPI) topics, field visits (Nigeria) (36)	Inner setting, characteristics of individuals, process	Penetration	Enable rapid increase of human resource capacity
Promote supervision *(n = 20)*	Provide ongoing supervision focusing on the intervention	• Using geographic information system (GIS) tracking to monitor vaccination team activity (Nigeria) (47) • Using LQAS evaluation to verify supervisory checking during NIDs (Pakistan) (48)	Inner setting, process	Fidelity, penetration, coverage	Identify missed targets and ensure accountability; identify opportunities for increased supportive supervision
Involve experts on management and use of data generated *(n = 12)*	Involve, hire, and/or consult experts to inform management on the use of data generated by implementation efforts	• Conducting joint evaluation by government and technical partners to evaluate program implementation (PAHO Region) (49)	Inner setting, process	Coverage, fidelity	Encourage adoption of new national strategies to improve implementation of global program
Shift and revise roles of providers *(n = 4)*	Shift and revise roles among professionals who provide care, and redesign job characteristics	• Engaging medical college interns and social workers to conduct house-to-house follow-up with families resistant to OPV (India) (50)	Characteristics of Individuals	Acceptability	Improve uptake by engaging community members with trusted source of information (i.e., interns from medical college perceived as favorable compared to district hospital staff where quality is low)
Learn from experts *(n = 4)*	Provide ways for individuals to directly observe experienced people engage with the targeted practices	• Building Emergency Operations Centers (EOC) at national and state levels to provide strategic direction, create data dashboards, analyze data, develop communication strategies, and monitor field activities (Nigeria) (51) • Deploying thousands of international consultants, including GIS experts who trained Nigerian health workers to use GIS for microplanning and to improve fieldworkers’ tracking (Nigeria) (47) • Training of Village Polio Volunteers (VPV) by polio eradication staff on AFP surveillance and community awareness (Somalia) (52)	Characteristics of individuals, inner setting	Coverage, penetration, timeliness	EOCs provided feedback to government officials and improved performance and accountability. e.g., data analysis helped to identify high-risk LGAs for prioritization of polio eradication activities Capacity building in new technology contributed to improved planning, logistics support and implementation Active community surveillance was conducted by VPVs to improve incident case finding in their local communities
Conduct workshops (to educate, provide feedback, iterate, etc.) *(n = 33)*	Hold meetings and workshops targeted toward different stakeholders	• Conducting interpersonal communication (ITP) and mother's meetings between SIAs to address misconceptions and fears (India) (53) • Conducting workshop to understand and address differences in data management processes for immunization dashboards (AFRO Region) (54)	Characteristics of individuals, process	Coverage, fidelity, acceptability	Addresses potential barriers to uptake; improves standardization of use of tools

^a^ In Tables 2–5, the number of articles that reported on a given implementation strategy (column 1), the “action target” (column 4) and “implementation outcomes affected” (column five) reflect analysis results from all included studies. Other domains, e.g., “how operationalized” (column three) and “justification” (column six) reflect results from 1 to 2 representative studies, selected to describe how and why implementation strategies were utilized within the polio eradication initiative.

**Table 5:** Implementation strategies: communications & advocacy^a^.

Implementation strategy	Definition	How operationalized *(actor, action, dose, temporality)*	Action target *(CFIR)*	Implementation outcomes affected^a^	Justification
Use mass media *(n = 31)*	Use media to reach large numbers of people to spread the word about the clinical innovation	• Utilizing radio and television messages to promote participation in National Immunization Days (NIDs) (Ghana) (55) • Forming of the Journalists Initiatives on Immunization Against Polio to develop communications aimed at highlighting immunization importance (Nigeria) (56)	Characteristics of individuals	Coverage, acceptability	Higher participation among those who received media messages
Identify and prepare champions and early adopters *(n = 20)*	Identify and prepare individuals who dedicate themselves to supporting, marketing, and driving through an implementation, overcoming indifference or resistance that the intervention may provoke in an organization	• Selecting volunteer community mobilizers who were religious or community leaders or household heads to serve on dedicated mobile teams (Nigeria) (27) • Involving relevant stakeholders (teachers in Qur’anic schools, *Ardos*, civil society leaders) as liaisons with the community (Nigeria) (16)	Outer setting, intervention characteristics	Coverage, acceptability, penetration	Improved tracking and service coverage of OPV and RI, including in persistently poor-performing districts
Increase awareness among the population *(n = 58)*	Increase population awareness of health interventions through various dissemination activities	• Leveraging various media to create awareness during mass polio campaigns depending on the sociocultural and economic contexts. In urban areas and urban slums television and loudspeakers (India) (57), market leaders and transportation officials (Nigeria) (46) were used; in rural areas, mosque announcements and loudspeakers (Pakistan) (58). • Developing a SMNet, deploying community mobilizers to raise awareness and accompany vaccinators at the household level, educating caregivers on polio immunization in non-campaign seasons, conducting “polio classes” for eligible children, and persuading non-vaccinated families on benefits of polio vaccine (India) (41)	Inner setting, intervention characteristics	Coverage, acceptability, effectiveness	Without awareness creation activities, caregivers were unaware of the mass campaign, and this was cited as one of the main reasons for under vaccination

^a^ In Tables 2–5, the number of articles that reported on a given implementation strategy (column 1), the “action target” (column 4) and “implementation outcomes affected” (column five) reflect analysis results from all included studies. Other domains, e.g., “how operationalized” (column three) and “justification” (column six) reflect results from 1 to 2 representative studies, selected to describe how and why implementation strategies were utilized within the polio eradication initiative.

**Table 6:** Implementation outcomes.

Outcome type	Operational definition of outcome	Type of measure	Measure (CI)	Direction	Study design	Controls	Implementation strategies utilized	Article
Acceptability	Percent relative change in children vaccinated per day at transit sites in India within intervention districts following inclusion of Muslim members on transit teams an increased number of transit sites	%	18,194 *(pre)* → 21,588 *(post)* (18.7% increase) *Comparison group:* 16,449 *(pre)* → 14,887 *(post)* (9.5% decrease)	Improvement	Longitudinal or pre/post test	Non-equivalent	Change service sites to increase access; Build robust record system to capture outcomes; Centralize assistance for implementation issues; Develop mechanisms for feedback, and monitoring and evaluation; Shift and revise roles of providers; Make training dynamic and varied	(59)
	Percentage of missed children following youth engagement strategy to improve acceptability of polio immunization coverage among previously non-compliant households in Nigeria.	%	11.6% (6.6–16.6) → 7.9% (2.3–13.5)	Improvement	Longitudinal or pre/post test	None	Identify or build a dissemination organization; Centralize assistance for implementation issues; Develop mechanisms for feedback, and monitoring and evaluation; Involve stakeholders, workers, and consumers in the implementation effort; Leverage existing collaborations and networks; Capture and share local knowledge, opinions, and needs; Recruit, designate, and train leaders	(22)
Adoption	Odds of being vaccinated based on prior awareness of the campaign following a household-based awareness campaign	Odds ratio	6.8 (5.6–8.3) → 6.4 (4.4–9.4)	No change	Longitudinal or pre/post test	None	Increase awareness among the population; Identify and prepare champions and early adopters	(60)
Cost	Total savings of GPEI over period of 55 years (1986–2040), assessing costs of treatment, rehabilitation, and vaccination with costs of eradication program	USD	$13.64M saved	Improvement	Modeling	None	Model and simulate desired changes and outcomes	(61)
	Annual expenditure (USD thousands) on polio eradication in Bangladesh between 1994 and 1997	USD	$7,104,000 expended	Improvement	Longitudinal or pre/post test	None	Acquire additional funding to facilitate implementation; Adapt physical structures and equipment to interventions	(62)
	Annual expenditure (USD thousands) on polio eradication in Cote d'Ivoire between 1996 and 1998	USD	$2,009,000 expended	Improvement	Longitudinal or pre/post test	None	Acquire additional funding to facilitate implementation; Adapt physical structures and equipment to interventions	(62)
	Percent of total funding requirements locally mobilized funds for polio eradication implementation [defined as funds mobilized by the World Health Organization (WHO) country office including those from the Federal Government, and bilateral and multilateral grants] in Nigeria, comparing 2008–2011 to 2012–2015	USD	31% → 70%	Improvement	Longitudinal or pre/post test	None	Develop a formal implementation blueprint; Acquire additional funding to facilitate implementation; Leverage existing collaborations and networks	(63)
Fidelity	Percent of Acute Flacid Paralysis (AFP) cases negative for wild poliovirus (WPV) and vaccine-derived poliovirus (VDPV) that had inadequate stool and a follow up exam after paralysis onset in the Democratic Republic of the Congo (DRC) (target = 80%)	%	10% → 73%	Improvement	Longitudinal or pre/post test	None	Build robust record system to capture outcomes	(64)
	Surveillance index rate of AFP cases with two stool specimens collected within 14 days of the onset of paralysis (from 0.0- 1.0)	Index	0.51 → 0.92	Improvement	Longitudinal or pre/post test	None	Build robust record system to capture outcomes	(65)
	Proportion of late AFP cases with follow-up report submitted within 90 days of onset of paralysis	%	67% → 88%	Improvement	Longitudinal or pre/post test	None	Build robust record system to capture outcomes	(23)
	Stool adequacy rate at the national level	%	88% → 93%	Improvement	Longitudinal or pre/post test	None	Build robust record system to capture outcomes; Develop mechanisms for feedback, and monitoring and evaluation	(23)
	Non-polio AFP rate at the national level in cases per 100,000 children under 15 years of age	Count per 100,000 population	2.7 → 3.2	Improvement	Longitudinal or pre/post test	None	Build robust record system to capture outcomes; Develop mechanisms for feedback, and monitoring and evaluation	(23)
	Proportion of cases completely reported as a measure of the sensitivity of the polio surveillance system in India, comparing 1981–1992	%	8% → 32%	Improvement	Longitudinal or pre/post test	None	Build robust record system to capture outcomes; Develop mechanisms for feedback, and monitoring and evaluation	(66)
Penetration	Number of newly identified settlements through use of revised microplanning tool	Count	20,338 → 28,074	Improvement	Longitudinal or pre/post test	None	Involve stakeholders, workers, and consumers in the implementation effort; Involve experts on management and use of data generated; Build robust record systems to capture outcomes; Develop mechanisms for feedback, and monitoring and evaluation	(33)
	Proportion of children with non–polio-associated AFP who received ≥4 oral poliovirus vaccine (OPV) doses	%	80% → 90%	Improvement	Cross-sectional	None	Offer incentives or disincentives to providers and consumers; Identify and prepare champions and early adopters; Increase awareness among the population	(16)
	Proportion of children with no–polio-associated AFP who received Zero OPV doses	%	3% → 1%	Improvement	Cross-sectional	None	Offer incentives or disincentives to providers and consumers; Identify and prepare champions and early adopters; Increase awareness among the population	(16)
	Reduction in the number of unimmunized children with additional polio program staff deployed in high-risk polio states.	Count	1,298,442 → 117,149	Improvement	Longitudinal or pre/post test	None	Assess organizational ability and readiness	(36)
Coverage	Difference in count of nomadic children 0–59 months vaccinated with OPV after intervention from baseline between intervention to comparison districts	Count	10,275 *(pre)* 24,032 *(post)* *Comparison group:* 20,011 *(pre)* 18,381 *(post)*	Improvement	Longitudinal or pre/post test	Non-equivalent^a^	Develop mechanisms for feedback, M&E Build robust record systems to capture outcomes; Centralize assistance for implementation issues; Promote supervision	(38)
	Proportion of unvaccinated children at street intersection transit sites, comparing beginning to end of Supplemental Immunization Activity (SIA)	%	3 → 24%	Improvement	Longitudinal or pre/post test	Non-equivalent	Count of children vaccinated with OPV at mass transit sites	(59)
	% children 0–59 months who received <3 RI OPV doses (pre-mass campaign) vs. % of children 0–59 months who received 2 OPV doses during two mass campaigns	%	68.90% *(pre)* *93.40% (post)*	Improvement	Longitudinal or pre/post test	None	Build robust record systems to capture outcomes; Develop mechanisms for feedback, monitoring and evaluation; Increase awareness among the population; Use mass media	(67)
	Proportion of children <5 vaccinated at transit stops among all children vaccinated by 3 Local Government Areas (LGAs) in Nigeria	Proportion	87,502 children vaccinated at transit sites/2,781,162 total children vaccinated by the 3 LGAs (3.2%). The 87,502 children represented a 138%–318% pre-post increase in the number of children vaccinated by the transit.	Improvement	Longitudinal or pre/post test	None	Involve stakeholders; Increase awareness; Recruit, designate, and train leaders; Promote supervision; Develop mechanisms for feedback, monitoring and evaluation	(46)
	Number of chronically missed settlements as an estimation of geographic coverage by polio vaccination teams	Count	5,833 (2014) → 1,257 (2015)	Improvement	Longitudinal or pre/post test	None	Promote supervision; Develop mechanisms for feedback, monitoring and evaluation	(68)
	Number of newborns receiving OPV0 from volunteer community mobilizers (VCMs) in six high-risk districts in Nigeria	Count	713,151 (2013) → 938,703 (2015)	Improvement	Longitudinal or pre/post test	None	Involve stakeholders; Identify and prepare champions and early adopters; Increase awareness among the population	(68)
	% of target population (children 0–59 months) receiving OPV3 via routine immunization systems in Anambra state, Nigeria (monthly)	%	21% (January 2010) → 74% (December 2010)	Improvement	Repeated cross-sectional surveys	None	Involve stakeholders, workers, and consumers in the implementation effort; Increase awareness among the population; Promote supervision; Develop mechanisms for feedback, monitoring and evaluation; Recruit, designate, and train leaders	(30)
	% population vaccinated with OPV during SIAs (effect of SIA on OPV coverage)	%	95.6% (2013) → 100.8% (2015)	Improvement	Repeated cross-sectional surveys	None	Involve stakeholders, workers, and consumers in the implementation effort; Increase awareness among the population; Adapt physical structures and equipment to interventions; Develop mechanisms for feedback, monitoring and evaluation	(21)
	Proportion of children vaccinated in polio booths during National Immunization Days (NIDs) in one locality in South Delhi, India following an Information, Education, and Communication (IEC)	Proportion	39% → 87%	Unknown	Longitudinal or pre/post test		Involve stakeholders, workers, and consumers in the implementation effort; Increase awareness among the population	1,462

^a^ Pre-post assessment was only done at the intervention site (and not among non-equivalent comparison groups).

**Table 7:** Service outcomes.

Outcome type	Operational definition of outcome	Measure	Value (CI)	Direction	Study design	Controls	Implementation strategies utilized	Article
Timeliness	Percent of AFP cases with 2 stools collected less than 14 days after paralysis onset in DRC (target = 80%)	%	82% → 84%	Improvement	Longitudinal or pre/post test	None	Build robust record system to capture outcomes	(64)
	Duration in days from paralysis onset to notification of AFP cases by Village Polio Volunteers (VPVs), Somalia, 2014–2016	Mean	5.4 (4.84–5.97) → 3.73 (3.32–4.14)	Improvement	Longitudinal or pre/post test	None	Leverage existing collaborations and networks	(52)
	Duration in days from paralysis onset to notification of AFP cases from other sources, Somalia, 2014–2016	Mean	4.76 (4.32–5.21) → 3.82 (3.3–4.34)	Improvement	Longitudinal or pre/post test	None	Leverage existing collaborations and networks	(52)
Efficiency	Cold chain sickness rate, defined as the proportion of cold chain equipment out of order at any point of time	%	9.8% → 6%	Improvement	Longitudinal or pre/post test	None	Assess organizational ability and readiness; Adapt physical structures and equipment to interventions	(69)
	Proportion of wards with updated microplans as a measure of additional polio staff's contribution to microplanning in high-risk states	%	35% → 73%	Improvement	Longitudinal or pre/post test	None	Develop a formal implementation blueprint; Acquire additional funding to facilitate implementation; Build robust record systems to capture outcomes; Offer incentives or disincentives to providers and consumers; Develop mechanisms for feedback, and monitoring and evaluation; Visit other sites where similar efforts have been successful; Shift and revise roles of providers; Conduct workshops (to educate, provide feedback, iterate etc.); Make training dynamic and varied; Recruit, designate, and train leaders; Use train-the-trainer strategies; Promote supervision	(36)
	Percent of positive feedback received following introduction of systematic accountability framework to improve performance of the WHO Nigeria polio program staff	%	61% → 74%	Improvement	Longitudinal or pre/post test	None	Build robust record systems to capture outcomes; Develop mechanisms for feedback, monitoring, and evaluation	(70)
Effectiveness	Odds of being aware of polio campaign comparing households that did or did not receive a social mobilization visit in the days preceding the campaign	Odds ratio	16.9 (10.1–28.2)	Unknown	Longitudinal or pre/post test	None	Increase awareness among the population; identify and prepare champions and early adopters	(60)
	Proportion of household who were aware of the November 2013 immunization round after social mobilization activities took place	%	95.6%	Unknown	Longitudinal or pre/post test	None	Increase awareness among the population; identify and prepare champions and early adopters	(60)
	Non-polio AFP rate per 100,000 in children under 15 in Mpumalanga province, South Africa (WHO target = 1)	Rate	0.56 (0.2–1.21)	Improvement	Longitudinal or pre/post test	None	Build robust record systems to capture outcomes; recruit designate and train leaders	(71)
	Non-polio AFP rate per 100,000 as measure of additional polio staff's contribution to AFP surveillance in in high-risk polio states	Rate	0.098% → 0.226	Improvement	Longitudinal or pre/post test	None	Recruit, designate and train leaders; promote supervision	(36)
	Average state campaign effectiveness achieved in Kano, Nigeria. Campaign effectiveness was defined as the change in reported OPV doses by the number of SIA linked to change in immune fraction by OPV serotype	Percentage	35% (30–41%) (2013)→ 75% (64–86%) (2014)	Improvement	Modeling	None	Develop mechanisms for feedback, monitoring, and evaluation	(25)
	Percent efficacy of monovalent OPV against Type 1 polio in Nigeria	%	67% (39%-82%)	Unknown	Case-control	Non-equivalent	Model and simulate desired changes and outcomes; Develop mechanisms for feedback, and monitoring and evaluation	(72)
	Percent efficacy of trivalent OPV against Type 3 polio in Nigeria	%	18% (9%-21%)	Unknown	Case-control	Non-equivalent	Model and simulate desired changes and outcomes; Develop mechanisms for feedback, and monitoring and evaluation	(72)
	Number of mosque announcements as a potential determinant of the difference in percent of “X households” (unvaccinated) converted to “P households” (vaccinated against polio) between Community Mobilization Coordinators (CMC) controlled and non-CMC controlled areas of a block	Coefficient	3.28 (0.02–6.58)	Improvement	Dose response	Non-equivalent	Change service sites to increase access; Identify or build a dissemination organization; Develop mechanisms for feedback, and monitoring and evaluation; Involve stakeholders, workers, and consumers in the implementation effort; Identify and prepare champions and early adopters; Increase awareness among the population	(73)
	Number of Bullawa Tollies (child mobilizers) as a potential determinant of the difference in percent of X households converted to P between CMC controlled and non-CMC controlled areas of a block	Coefficient	0.15 (−1.47–1.77)	Improvement	Dose response	Non-equivalent	Change service sites to increase access; Identify or build a dissemination organization; Develop mechanisms for feedback, and monitoring and evaluation; Involve stakeholders, workers, and consumers in the implementation effort; Identify and prepare champions and early adopters; Increase awareness among the population	(73)
	Non-polio AFP rate in children under 15 years per 100,000 as measure of AFP surveillance system	Rate	4.5 → 6.4	Improvement	Longitudinal or pre/post test	None	Build robust record system to capture outcomes	(74)
Equity	Percent of total population of Balochistan/FATA, Pakistan persistently under vaccinated comparing 2008–2010–2011	**%**	34.2% (28–40.6) → 34.2% (28–40.6)	No change	Modeling	None	Develop mechanisms for feedback, and monitoring and evaluation	(75)

**Table 8:** Impact outcomes.

Outcome type	Operational definition of outcome	Measure	Value (CI)	Direction	Study design	Controls	Implementation strategies utilized	Article
Morbidity	Proportion of wild poliovirus (WPV) positive environmental samples tested for poliovirus in Karachi, Sindh, Pakistan at KHI-GI-Chakora Nulla collection site in 2011 vs. 2013	Proportion	6/12 → 0/10	Improvement	Longitudinal or pre/post test	None	Build robust record system to capture outcomes	(76)
	Proportion of WPV-positive environmental samples tested for poliovirus in Lahore, Punjab, Pakistan at LHR-Gulshan-e-Ravi Station collection site in 2011 vs. 2013	Proportion	5/12 → 0/10	Improvement	Longitudinal or pre/post test	None	Build robust record system to capture outcomes	(76)
	Percent of samples positive for WPV1 among all samples collected in 4 provinces of Pakistan - Sindh, Punjab, Khyber Pakhtun Kwa, Bauchistan	%	40%	Unknown	Longitudinal or pre/post test	None	Build robust record system to capture outcomes	(76)
	Number of confirmed wild poliovirus cases polio cases in Nigeria, comparing 2012–2013	Count	122 → 53	Improvement	Longitudinal or pre/post test	None	Build robust record system to capture outcomes; Develop mechanisms for feedback, and monitoring and evaluation	(51)
	Number of AFP cases notified by health authorities to regional WHO office and to lab, defined as children under 15 with AFP illness	Count	3→12	Improvement	Longitudinal or pre/post test	None	Build robust record system to capture outcomes	(65)
	Number of paralytic polio cases in children aged 0–59 months at a hospital in Kano, Northwest Nigeria in 2007 vs. 2016	Count	16→305	Deterioration	Cohort	None	Develop mechanisms for feedback, and monitoring and evaluation	(77)
	Confirmed cases of poliomyelitis infection in Lao People's Democratic Republic per official AFP surveillance data, comparing 1990–1996	Count	18 → 3	Improvement	Longitudinal or pre/post test	None	Build robust record system to capture outcomes; Develop mechanisms for feedback, and monitoring and evaluation	(78)
	Incidence of poliomyelitis per 100,000 based on household surveys and routine surveillance in India	Rate	25 → 6.3	Improvement	Longitudinal or pre/post test	None	Build robust record system to capture outcomes	(66)
	Rate per 100,000 of children paralyzed due to poliomyelitis, comparing 1989–1991	Rate	4.4 → 1.5	Improvement	Longitudinal or pre/post test	None	Develop mechanisms for feedback, and monitoring and evaluation	(79)
	Number of confirmed polio cases in Pakistan, comparing 2001 and 2009	Count	119 → 144	Deterioration	Longitudinal or pre/post test	None	Develop mechanisms for feedback, and monitoring and evaluation	(18)
	Number of children with acute paralytic poliomyelitis admitted to the SAT Hospital in Trivandrum in Kerala Statecomparing 1986–1987		119 → 458	Deterioration	Longitudinal or pre/post test	None	Develop mechanisms for feedback, and monitoring and evaluation	(80)
	Number of WPV cases as a measure of GPEI's impact on rapid response and control of disease outbreaks in Africa	No.	122 → 6	Improvement	Longitudinal or pre/post test	None	Identify or build a dissemination organization; Develop mechanisms for feedback, and monitoring and evaluation; Conduct workshops (to educate, provide feedback, iterate etc.); Capture and share local knowledge, opinions, and needs	(81)
Mortality	Mortality ratio of children under 5, comparing no polio vaccine to 1–2 doses of OPV	Ratio	0.46 (0.18–1.15) *Comparison:* 0.67 (0.48–0.94)	Improvement	Longitudinal or pre/post test	Non-equivalent	Adapt physical structures and equipment to interventions	(82)
	Mortality ratio of children aged 0–5 months comparing no polio vaccine to 1–2 doses of OPV	Ratio	0.13 (0.02–0.68) → *Comparison:* 0.56 (0.31–1.01)	Improvement	Longitudinal or pre/post test	Non-equivalent	Adapt physical structures and equipment to interventions	(82)

Study publication dates ranged from 1989 to 2018, but the majority (*n* = 80, 52.98%) were published between 2014 and 2018, coinciding with the fifth GPEI Strategic Plan (2013–2018) (83). Articles were relevant to multiple WHO regions covering a large swath of LMICs, especially the African (AFR), Americas (AMR), Eastern Mediterranean (EMR), and South-East Asia (SEAR) regions, though there was a clustering of articles in countries that remained polio endemic in 2020 or were focus LMICs for the GPEI [e.g., Nigeria (*n* = 50), India (*n* = 45), Pakistan (*n* = 22), Ethiopia (*n* = 12), and Democratic Republic of Congo (*n* = 8)]. The lack of articles from the WPR region likely reflect that the region was declared polio free in 2000 (84). Because the studies assessed a wide array of implementation strategies and their outcomes relevant to polio eradication, study samples were varied, however, most were focused on children 0–59 months, which is the target age range for three doses of poliovirus immunization. Of the included studies (*n* = 152), most (*n* = 135) utilized only quantitative methods, drawing heavily from surveys (*n* = 82, including both household and other surveys) and health management information system (HMIS) data (*n* = 49). We also included 17 studies which used a mixed methods approach and included qualitative methods such as focus group discussions and key informant interviews. Notably, the majority of studies reviewed utilized an adequacy design, that is, were cross-sectional in nature and did not include equivalent or non-equivalent comparison groups. Characteristics of the included studies are described further in Table 1.

### Implementation strategies

Tables 2–5 describe the implementation strategies utilized in the global polio eradication initiative from 1988 to 2018 following four themes identified from the broader STRIPE scoping review: management and problem solving (7 strategies referenced 106 times out of 496 total strategy references); monitoring and evaluation (4 strategies referenced 75 times out of 496 total references); engagement and capacity building (12 strategies referenced 206 times out of 496 total references); and communications and advocacy (3 strategies referenced 109 times out of 496 total references). A majority of included articles (*n* = 127, 83.6%) reported mostly multifaceted (i.e., combined multiple strategies or components), with an average of four implementation strategies (95% CI: 3.6, 4.7) reported on, and only 25 articles (16.4%) reported a single strategy. Across all themes, the most frequently documented implementation strategies were developing mechanisms for feedback, monitoring, and evaluation (69 out of 152 articles, 45.4%); increasing awareness among the population (58 out of 152 articles, 38.2%); involving stakeholders, workers, and consumers in the implementation efforts (46 out of 152 articles, 30.3%); conducting workshops (33 out of 152 articles, 21.7%); using mass media (31 out of 152 articles, 20.4%); and building robust record systems to capture outcomes (31 out of 152 articles, 20.4%). The most common implementation outcomes affected by these strategies were coverage (81% of strategies), acceptability (50% of strategies) and fidelity (46% of strategies). Conceptual definitions and operational examples for each of the implementation strategies are provided, along with explanations for how each strategy was used in the GPEI in Tables 2–5.

### Implementation, service delivery, and impact outcomes

Tables 6–8 describe the implementation, service delivery, and impact outcomes that were described in the 43 unique studies included in the outcome analyses (see Figure 1: PRISMA flow diagram), that is studies that included data collected at multiple timepoints and/or control or comparison groups. There were 66 outcomes extracted from these 43 unique studies. Out of the 66 outcomes, coverage (*n* = 13) and morbidity (*n* = 12) were the most frequently identified outcomes, followed by effectiveness (*n* = 9) and fidelity (*n* = 6). Longitudinal or pre/post studies were the most frequently employed study design for assessing the influence of implementation strategies on the outcomes (*n* = 46) followed by cross-sectional data collection (*n* = 11). For the majority of outcomes (*n* = 57), there was no comparison group. Most studies reported changes in outcomes over time. Improvement in outcomes were reported in most cases (*n* = 44), whereas only 5 outcomes were reported as worse than expected over the course of the study.

Of the 32 implementation outcomes extracted, the most frequently described outcomes were related to coverage (Table 8). Although the operational definition for coverage varied, these studies generally reported on the proportion of children that were vaccinated within a geographic area. The operational definitions for other implementation outcomes captured elements of other GPEI program components. For example, fidelity outcomes largely reported on the processes related to AFP surveillance systems, acceptability outcomes tracked the impact of community engagement strategies, while cost outcomes reflected on overall program expenditures. For most implementation outcomes, it was difficult to identify influential implementation strategies because of the multifaceted nature of most of the strategies and limited description on how they were specified. However, all 6 of the fidelity outcomes were influenced by strategies that build robust record systems to capture outcomes. Notably, there were no included studies that reported on outcomes related to appropriateness, feasibility, or sustainability.

Among the service delivery outcomes, effectiveness was the most frequently reported outcome (Table 7). Effectiveness outcomes focused on a range of issues, from the effectiveness of social mobilization campaigns to the efficacy of various polio vaccinations, and to the overall effectiveness of GPEI programming in geographic areas. Most of the timeliness outcomes related to the speed at which the AFP surveillance system found and reported suspect cases of polio. An example of an efficiency outcome is the proportion of wards (sub-districts) using updated microplans in high-risk states. Equity concerns were only directly addressed by two modeling studies. There were no included studies that reported on patient safety, or the level of patient-centered care provided by GPEI programs.

Outcome measures of morbidity were reported more frequently than mortality (Table 8). Morbidity outcome measures largely captured the incidence or prevalence of polio within a population. Across the 12 morbidity outcomes recorded, 6 assessed for implementation strategy on building robust record systems, 4 were assessed for developing mechanisms for feedback, and monitoring and evaluation, and 2 outcomes were assessed for both implementation strategies. Only one study examined the polio mortality over time and found a beneficial impact of supplementary immunization activities (e.g., house-to-house, mobile posts, and hotspots vaccination campaigns) on mortality ratios in children even during conflict.

## Discussion

### Synthesis

The global polio eradication initiative is one of the largest public health initiatives in the world (4)—and provides important lessons in implementation research and practice for improving delivery of health programs and services globally (6). In this paper, we examined implementation strategies and outcomes that were used for facilitating polio vaccination at different socioecological levels and diverse settings using a theory-based and systematic approach drawing heavily from theories, models, and frameworks in implementation science. We found that most implementation strategies deployed under GPEI in LMICs were multifaceted, focusing on stakeholder engagement and capacity building, and addressing management and problem solving in real time. These strategies were only weakly associated with implementation outcomes, especially coverage and fidelity, and service delivery and impact outcomes.

Whereas the ERIC framework (3) was helpful to initially organize the implementation strategies described as part of the GPEI, we had to adapt it to accommodate other strategies which were salient for the LMIC settings, but not described in any categories of the ERIC framework. For example, strategies focused on setting up and adapting infrastructure for services delivery, developing community partnership and community-led engagement activities (Tables 2–5) featured prominently and were relevant across different LMIC settings reviewed. This fact may point to the limitation of the ERIC framework in that it was originally designed for categorizing implementation strategies for supporting clinical interventions (and not population-based, public health interventions), from a high income country perspective, and lacks sufficient coverage of health systems strengthening strategies which are often necessary in resource-limited settings, especially in LMICs, to facilitate effective implementation and impact of evidence-supported interventions.

Indeed, implementation strategies deployed in low-resource settings need to be coupled with other strategies and efforts to build and strengthen health systems. For instance, some of the most frequently applied implementation strategies described for supporting the delivery of the polio vaccines in this review (e.g., developing mechanisms for feedback, increasing health awareness among population, building robust record systems to capture outcomes) were coupled with additional health system strengthening strategies (e.g., building advanced laboratory systems and community-based surveillance) that were critical for providing essential health services more broadly in LMIC settings (84).

The findings from this review are consistent with the priorities of the polio eradication initiative and its operational emphases over time, reflecting priorities to engage communities and individuals, reach hard-to-reach and hard-to-vaccinate populations, and improve program operations (17–21, 84). Consistent with other studies, strategies to build partnership and coalitions (20, 84), co-deliver other interventions and provide other health services beyond polio vaccination (17), develop mechanisms for feedback and accountability (84), engage local community and gain trust (21), conduct monitoring and evaluation including setting up robust data system (84) were all identified in this review.

What this study adds that has not been explicitly considered in the other studies is the influence of these implementation strategies on implementation, service delivery, and impact outcomes which can facilitate an evaluation of the likelihood of these strategies to achieve their expected results when applied to other programs (Tables 6–8)—thus, facilitating decision-making and prioritization efforts around these implementation strategies. Coverage of polio vaccination was predictably the central measure of the global polio eradication initiative. Hence, most of the implementation strategies reviewed were positively linked to these implementation outcomes. Two other implementation outcomes—acceptability and fidelity—also emerged from the data as significant for driving global health services delivery as demonstrated by the influence of strategies to build robust record system and develop mechanisms for feedback on these outcomes. The emphasis on fidelity was strong throughout the initiative, reflecting the top-down and central-command approach of the GPEI, and a response to limited health infrastructure and capacities in many of the implementing environments. The top-down and central-command approach was also reflected in the initiative's data-driven approach to planning and implementation, which leveraged strategies geared toward health information systems (e.g., building robust record systems to capture outcomes, developing mechanisms for feedback, monitoring, and evaluation).

Studies examining fidelity as an outcome of interest also described the initiative's investment in deploying human resources for health for polio-related activities (e.g., recruiting health workers, making training dynamic and varied, promoting supervision). Over time, acceptability became an increasing concern for the GPEI as implementer's struggled to penetrate pockets of low coverage and faced resistance from communities who were fatigued or mistrustful of the campaign (85). As has been well documented, the polio eradication initiative was compelled to address these issues through numerous engagement and communications strategies (e.g., identifying and preparing champions and early adopters, leveraging existing networks and collaborations), often tailored to meet highly localized needs. The polio eradication experience suggests that achieving coverage of health interventions is dually dependent on implementation processes that enable both precision and modification, and attention to demand-side factors that affect uptake and satisfaction.

Given the unique nature of the GPEI (a well-described evidence-based intervention in the polio vaccine, an ambitious eradication goal which drove the perception that an urgent response was warranted, a massive influx of resources, and an expectation of a discrete timeline), it is not altogether surprising that appropriateness, feasibility, and sustainability were rarely studied implementation outcomes. However, the absence of attention to sustainability has borne out over time to be an issue as implementers continue to struggle with how to integrate polio activities with other service delivery priorities and integrate programmatic assets into the broader health infrastructure and health system (86). Future efforts would benefit from developing and evaluating strategies to improve sustainability of health interventions. With regards to services delivery outcomes, the focus of reported studies on timeliness and speed of program delivery, and lack of attention to equity, are noteworthy given the ongoing and intractable challenges to reach marginalized populations under the GPEI. This provides important lessons for global vaccine delivery programs aimed at addressing pandemics and adequately responding to changing infectious disease dynamics. Speed and equity are not mutually exclusive goals.

Our synthesis revealed a few significant gaps in the literature which warrant commentary. First, throughout the literature implementation strategies were poorly described and, importantly, were not explicitly tied to implementation, service delivery, or impact outcomes. Indeed, there seemed to be a division in the literature between manuscripts which described polio eradication strategies in-depth, and those that measured polio-relevant outcomes, but which were only loosely connected to specific eradication strategies. This may partially reflect an operational reality that implementation strategies are pursued simultaneously, and researchers may have struggled to describe and measure the relationship between implementation strategies. Programmatic information systems and internal reports may better capture these dynamics, however, the utility of those findings for assessing implementation strategy effectiveness is limited if they are not cogently shared with a wider audience. Second, as was noted, a very limited set of articles included in the review demonstrated no change or a deterioration in the outcome of interest. As a result, the literature provides limited insights into those implementation strategies that were attempted and failed. This may reflect a larger trend in public health literature to focus publications on proven solutions (87), and in this context, a need to disseminate learnings which may have wider applicability. Efforts to document failures may have a critical role to play in building learning health systems that can refine programs and policies in real time as they adapt to changing conditions.

### Strengths and limitations

Our study presents a comprehensive examination of implementation strategies leveraged throughout the polio eradication effort, drawing from a large sample of peer-reviewed articles. While there have been many efforts to document program strategies (88–90) few studies have described implementation strategies with the operational detail we present here. By utilizing standardized definitions for implementation strategies (3) and following operational guidelines for elaborating on them (8) we have tried to make our results interpretable and enable their practical use, while also contributing to the relevant theories, models and frameworks from the field of implementation science. For example, we realized the need to further organize implementation strategies around a context/domain-specific strategy taxonomy to complement the ERIC framework as shown in Tables 2–5 (i.e., framing implementation strategies around broader implementation objectives that they aim to accomplish, e.g., management and problem-solving, monitoring and evaluation, engagement and capacity building, communication and advocacy) to guide readers in understanding the context surrounding the deployment of specific strategy in different countries. This additional framework may be useful for guiding the choice of implementation strategies, especially in settings where a strategy may not necessarily be named and formulated as described by the ERIC framework—and enhance the contextual generalizability of strategies in implementation analysis (e.g., while two strategies may have different names in different settings, the lessons surrounding their implementation may be generalizable given any similarities of implementation objectives in the different settings). Hence, we recommend that this additional framework be used for naming strategies alongside the ERIC framework where feasible. In our analysis, we have taken an iterative, theory-based approach, ensuring high inter-rater reliability among our analysts. As far as we know, the combining of the ERIC and CFIR frameworks as operationalized for analyzing implementation strategies may be novel to this study.

Still, our study is not without limitations. The data itself presented numerous challenges which limited the depth of quantitative analysis we were able to conduct. Many studies were missing sample size information, while others did not provide denominators for outcomes measured. This made it challenging to evaluate the effectiveness of various strategies and prohibited conducting a meta-analysis. Additionally, this study centers on research evaluating quantitative measures; there is, however, qualitative work evaluating implementation strategies in this space—and the synthesis of these works could be the focus of future studies. Despite these limitations, our review of quantitative analyses provides a unique synthesis of how, when, and to what effect implementation strategies have been deployed throughout the course of the GPEI and have also pointed to clear gaps in how implementation outcomes are measured and reported. Notably, because our review was part of a larger study, it only includes articles through 2018. The included articles reflect a period of high intensity for the GPEI and are thus rich for understanding the implementation strategies used, however, future studies may wish to update these findings to explore any new innovations that have come in the final periods of the GPEI, particularly as the GPEI has turned to focus on integration into essential health systems (91). Finally, our analysis focused on peer-reviewed literature published in English; this provided helpful insights into how implementation strategies and outcomes are reported but may have led to the exclusion of some findings only available in the grey literature or published in other languages.

### Implications for future research and practice

Within global health service delivery, more can and should be done to link the measurement of implementation strategies utilized in programs like the polio eradication effort to implementation, service delivery, and impact outcomes, and to evaluate those pathways in depth. These strategies should be evidentially or theoretically linked to specific implementation barriers or facilitation levers to define their objectives. While we were unable to conduct a full meta-analysis, our study did identify specific implementation strategies (Tables 6–8) that demonstrated a positive effect on implementation outcomes such as acceptability, fidelity, and coverage. By describing these in full, implementers can assess the appropriateness of these strategies (for example, changing service sites to increase access and acceptability, and involving stakeholders in the implementation effort to improve penetration) to their initiatives, and better drive outcomes.

As these implementation strategies are taken up in future initiatives, they should be coupled with embedded implementation research efforts to answer critical questions in real-time which can inform program adaptation and provide further insights into strategy effectiveness and contribute to broader health systems resilience (92). These studies should consider methodological instruments which enable the evaluation of both individual and combined implementation strategies, and their mechanisms of action. They should also emphasize the measurement of implementation outcomes which provide valuable information as to implementation strengths and weaknesses across numerous dimensions affecting delivery, uptake, and sustained use of health interventions.

Critically, implementation strategies and outcomes must be measured in a *linked* way, and in consideration of influencing variables which impact implementation over time, and lead to programmatic and systemic adaptations. Multiple and mixed methods research, which were not commonly reported in this review, are one avenue for advancing our understanding in this regard. As others have previously demonstrated, mixed method designs allow for hypothesis testing, while also providing a deeper understanding of implementation mechanisms (9, 93). Advancing theory can also help address the gaps identified in this study. While many of the implementation strategies described by the ERIC framework were relevant to polio eradication, there were health system strengthening strategies (e.g., human resources for health innovations) that did not fit under the framework, and the orientation of this framework to high-income countries was a significant limitation as described in the discussion above. Research to validate the appropriateness of the implementation strategies included in the ERIC framework for low and middle-income settings, and to describe missing implementation strategies relevant to ongoing public health initiatives (e.g., disease control, primary health care) should be considered. Organizing these strategies according to specific implementation outcomes can also support the development of theoretically grounded monitoring and evaluation platforms to better assess the effectiveness of implementation strategies for achieving relevant health outcomes.

Finally, published evaluations of this nature should not shy away from presenting failures to improve public health outcomes. Instead, these studies should endeavor to explain *why* targets were unmet to facilitate understanding and inform future implementation. Neglecting to address these gaps risks the repeated selection of inappropriate, ineffective strategies which may be predicated on potentially incorrect assumptions and inconclusive evidence. Practitioners would benefit most from research that helps them to reliably determine the potential effectiveness of strategies, and to assess necessary adaptations for programmatic and contextual specificities.

## Conclusion

This review provides a catalogue of implementation strategies and outcomes relevant for global health services delivery drawing from the global polio eradication initiative through a systematic and theory-driven synthesis. Implementation strategies to develop mechanisms for feedback, increase awareness among population, engage communities and other stakeholders, and build robust record system to capture outcomes were found to be frequently applied across diverse settings with loose evidence on their positive influence on implementation, service, and impact outcomes. It is important to carefully consider the context in which these strategies and to consider coupling them with health system strengthening strategies (e.g., building health infrastructure) in resource-limited settings to maximize impact. This review advances theories in implementation science through the application of models and frameworks for operationalizing implementation strategies and outcomes, demonstrating the utility and gaps in using these models and frameworks for specifying strategies applied in LMIC settings. It demonstrates the gaps in the literature around the effectiveness and impact of implementation strategies relevant for global health services delivery and describes important lessons and guidance for achieving the goals of the GPEI and similar global health services delivery programs.

## Data Availability

The original contributions presented in the study are included in the article/**Supplementary Material**, further inquiries can be directed to the corresponding author.

## Supplementary material

Supplementary File S1 Scoping Review PubMed Search Terms.

Supplementary File S2 Inclusion/Exclusion Criteria.

Supplementary File S3 Results of inter-rater reliability statistics.

Supplementary File S4 PRISMA checklist.

Supplementary File S5 Bibliography of articles identified for data extraction.
