# Supplementary material for: Lessons from polio eradication: a synthesis of implementation strategies for global health services delivery from a scoping review
Source: Front Health Serv. 2024 Aug 7;4:1287554. doi: 10.3389/frhs.2024.1287554 (PMC11335730; doi:10.3389/frhs.2024.1287554)
Supplement: Supplementary File S3 — Results of inter-rater reliability statistics. [file Datasheet3.docx]

**Inclusion/Exclusion Criteria**

**Inclusion criteria:**

Include the article IF:

1. Article met inclusion criteria for GPEI scoping review

a. Article must address some aspect of GPEI that relates to how it has worked and what can be learned.

b. Must NOT be focused exclusively on high-income countries (per World Bank classifications).

AND

2. Article was categorized by review team as “scientific journal in a peer-reviewed journal” or “review article”

AND

3. Article includes quantitative measurement of one or more implementation OR service delivery OR impact-level outcomes, as per definitions provided below.

Language: This review includes articles reported in English.

Timing: This review covers articles from the period January 1, 1988 to the date the search was conducted April 25, 2018.

**Exclusion criteria:**

Exclude the article IF:

1. Article was excluded from GPEI scoping review.

OR

2. Article was categorized by review team as one of the following document types: editorial, periodic update, news article, policy and policy analysis or critique, protocol for research or program, SOPs, unpublished project document, other

OR

3. Article does not measure implementation, service delivery, or impact-level outcomes.

OR

4. Study uses qualitative methods only (as opposed to quantitative, mixed, or multi-methods)

Language: Non-English articles are not included in this review.

Timing: Publications prior to January 1, 1988 are not included in this review.

***Exclusion tag definitions:***

Excluded articles will be tagged to indicate the rationale for exclusion. These tags include:

- **No outcome** - article does not measure implementation, service delivery or impact-level outcomes that measure **effectiveness** of implementation strategies deployed for polio eradication.
- **Non-qualifying epi/seroprevalence** - epi / seroprevalence studies that do not include two time points OR comparison districts and are not linked to an implementation strategy should be excluded.
- **Non-qualifying modeling** - model has an outcome that varies by non-programmatic features (e.g., the migration patterns of people on prevalence) as opposed to an outcome that varies by different implementation scenarios/strategies
- **Qualitative methods only** - article excluded because study utilized qualitative methods exclusively with no quantitative outcome measurements
- **Article type misclassified** - article is not a peer-reviewed scientific publication (e.g., it is an op-ed or editorial)
- **Not relevant** - article should not have been included in GPEI scoping review based on scoping review inclusion/exclusion criteria (LMICs, GPEI-related, after 1988, English)
