# Supplementary material for: Lessons from polio eradication: a synthesis of implementation strategies for global health services delivery from a scoping review
Source: Front Health Serv. 2024 Aug 7;4:1287554. doi: 10.3389/frhs.2024.1287554 (PMC11335730; doi:10.3389/frhs.2024.1287554)
Supplement: Supplementary File S4 — PRISMA checklist. [file Datasheet4.docx]

**Results of inter-rater reliability statistics**

This brief report summarizes the results of the inter-rater reliability tests for the study described in *Lessons from Polio Eradication: A* *synthesis of implementation strategies for global health services delivery from a scoping review*. The review comprised of 28 articles by 4 individual raters across 392 variables in the abstraction tool. Analyses were conducted in Stata (14.2).^1^

1. **Overall inter-rater reliability statistics**

**kappaetc v1-v111 , benchmark showscale**

**Interrater agreement Number of subjects = 392**

**Ratings per subject: min= 7**

**avg = 108.8**

**max = 111**

**Number of rating categories= 44**

**P cum. Probabilistic**

**Coef. Std.Err.P in. >95% [Benchmark Interval]**

**Percent Agreement 0.8623 0.0096 1.00 1.000 0.8000 1.0000**

**Brennan and Prediger 0.8591 0.0098 1.00 1.000 0.8000 1.0000**

**Cohen/Conger's Kappa 0.4781 0.0312 0.99 0.994 0.4000 0.6000**

**Scott/Fleiss' Kappa 0.5264 0.0270 1.00 1.000 0.4000 0.6000**

**Gwet's AC 0.8613 0.0097 1.00 1.000 0.8000 1.0000**

**Krippendorff's Alpha 0.5046 0.0294 1.00 1.000 0.4000 0.6000**

**Benchmark scale**

**<0.0000 Poor**

**0.0000-0.2000 Slight**

**0.2000-0.4000 Fair**

**0.4000-0.6000 Moderate**

**0.6000-0.8000 Substantial**

**0.8000-1.0000 Almost Perfect**

For all the 392 variables and across the multiple raters (we assume independence of raters and articles) for all articles, the overall percent agreement was **86%, implying almost perfect agreement** among raters based on the benchmark scale without accounting for agreement due to chance. The agreements that correct for chance ranged from 53% (Fleiss’ Kappa) to 86% (Gwet’s agreement coefficient). Gwet’s AC corrects for missing values, adjusts for the high agreement-low kappa paradox that is characteristic of Cohen’s and Fleiss’ kappa and incorporates the metric differences in Krippendorff’s alpha to accommodate different data types.^2,3^ It has been recommended for reporting the inter-rater reliability among multiple raters in health research as it provides more stable estimates.^4^ In this analysis, Gwet’s AC of 86% indicates overall almost perfect agreement among raters.^4^

1. **Inter-rater reliability statistics by article reviewed**

Some of the articles were selected randomly to evaluate agreement by article.

|  | Article #423 | | Article #523 | | Article #788 | | Article #927 | | Article #1274 | |
| --- | --- | --- | --- | --- | --- | --- | --- | --- | --- | --- |
|  | Coef | Probabilistic (Benchmark Interval) | Coef | Probabilistic (Benchmark Interval) | Coef | Probabilistic (Benchmark Interval) | Coef | Probabilistic (Benchmark Interval) | Coef | Probabilistic (Benchmark Interval) |
| Percent Agreement | **0.97** | **(0.80 – 1.00)** | **0.99** | **(0.80 – 1.00)** | **0.97** | **(0.80 – 1.00)** | **0.98** | **(0.80 – 1.00)** | **0.98** | **(0.80 – 1.00)** |
| Gwet’s AC | **0.97** | **(0.80 – 1.00)** | **0.99** | **(0.80 – 1.00)** | **0.97** | **(0.80 – 1.00)** | **0.98** | **(0.80 – 1.00)** | **0.98** | **(0.80 – 1.00)** |
|  | | | | | | | | |  |  |
|  | Article #1645 | | Article #1829 | | Article #1945 | | Article #2074 | | Article #2118 | |
|  | Coef | Probabilistic (Benchmark Interval) | Coef | Probabilistic (Benchmark Interval) | Coef | Probabilistic (Benchmark Interval) | Coef | Probabilistic (Benchmark Interval) | Coef | Probabilistic (Benchmark Interval) |
| Percent Agreement | **0.98** | **(0.80 – 1.00)** | **0.96** | **(0.80 – 1.00)** | **0.97** | **(0.80 - 1.00)** | **0.93** | **(0.80 – 1.00)** | **0.96** | **(0.80 – 1.00)** |
| Gwet’s AC | **0.97** | **(0.80 – 1.00)** | **0.96** | **(0.80 – 1.00)** | **0.97** | **(0.60 - 0.80)** | **0.93** | **(0.80 – 1.00)** | **0.96** | **(0.80 – 1.00)** |

**Benchmark scale**

**<0.0000 Poor**

**0.0000-0.2000 Slight**

**0.2000-0.4000 Fair**

**0.4000-0.6000 Moderate**

**0.6000-0.8000 Substantial**

**0.8000-1.0000 Almost Perfect**

Across articles, percent agreement of raters and the chance-corrected Gwet’s AC among raters ranged from 93% in article #2074 to 99% in article #523.

1. **Inter-rater reliability statistics by specific variables**
   1. **Q16 - strategies:** All articles reviewed were restricted to agreement on implementation strategies selected. Percent agreement of raters was perfect (1.0) across articles, except article #2074 where agreement was 0.75. The chance-corrected Gwet’s AC among raters also showed perfect agreement among raters and across articles, except article #2074 with 0.64 agreement.

|  | Article #423 | | Article #523 | | Article #788 | | Article #927 | | Article #1274 | |
| --- | --- | --- | --- | --- | --- | --- | --- | --- | --- | --- |
|  | Coef | Probabilistic (Benchmark Interval) | Coef | Probabilistic (Benchmark Interval) | Coef | Probabilistic (Benchmark Interval) | Coef | Probabilistic (Benchmark Interval) | Coef | Probabilistic (Benchmark Interval) |
| Percent Agreement | **1.00** | **(0.80 – 1.00)** | **1.00** | **(0.80 – 1.00)** | **1.00** | **(0.80 – 1.00)** | **1.00** | **(0.80 – 1.00)** | **1.00** | **(0.80 – 1.00)** |
| Gwet’s AC | **1.00** | **(0.80 – 1.00)** | **1.00** | **(0.80 – 1.00)** | **1.00** | **(0.80 – 1.00)** | **1.00** | **(0.80 – 1.00)** | **1.00** | **(0.80 – 1.00)** |
|  | | | | | | | | |  |  |
|  | Article #1645 | | Article #1829 | | Article #1945 | | Article #2074 | | Article #2118 | |
|  | Coef | Probabilistic (Benchmark Interval) | Coef | Probabilistic (Benchmark Interval) | Coef | Probabilistic (Benchmark Interval) | Coef | Probabilistic (Benchmark Interval) | Coef | Probabilistic (Benchmark Interval) |
| Percent Agreement | **1.00** | **(0.80 – 1.00)** | **1.00** | **(0.80 – 1.00)** | **1.00** | **(0.80 – 1.00)** | **0.75** | **(0.60 – 0.80)** | **1.00** | **(0.80 – 1.00)** |
| Gwet’s AC | **1.00** | **(0.80 – 1.00)** | **1.00** | **(0.80 – 1.00)** | **1.00** | **(0.80 – 1.00)** | **0.64** | **(0.60 – 0.80)** | **1.00** | **(0.80 – 1.00)** |

**Benchmark scale**

**<0.0000 Poor**

**0.0000-0.2000 Slight**

**0.2000-0.4000 Fair**

**0.4000-0.6000 Moderate**

**0.6000-0.8000 Substantial**

**0.8000-1.0000 Almost Perfect**

- 1. **Q42 - methods**

All articles reviewed were restricted to agreement on study methods selected. Percent agreement of raters was perfect (1.0) across articles, except article #2074 where agreement was 0.75. The chance-corrected Gwet’s AC among raters similarly showed perfect agreement among raters across articles, except article #2074 with 0.64 agreement.

|  | Article #423 | | Article #523 | | Article #788 | | Article #927 | | Article #1274 | |
| --- | --- | --- | --- | --- | --- | --- | --- | --- | --- | --- |
|  | Coef | Probabilistic (Benchmark Interval) | Coef | Probabilistic (Benchmark Interval) | Coef | Probabilistic (Benchmark Interval) | Coef | Probabilistic (Benchmark Interval) | Coef | Probabilistic (Benchmark Interval) |
| Percent Agreement | **1.00** | **(0.80 – 1.00)** | **1.00** | **(0.80 – 1.00)** | **1.00** | **(0.80 – 1.00)** | **1.00** | **(0.80 – 1.00)** | **1.00** | **(0.80 – 1.00)** |
| Gwet’s AC | **1.00** | **(0.80 – 1.00)** | **1.00** | **(0.80 – 1.00)** | **1.00** | **(0.80 – 1.00)** | **1.00** | **(0.80 – 1.00)** | **1.00** | **(0.80 – 1.00)** |
|  | | | | | | | | |  |  |
|  | Article #1645 | | Article #1829 | | Article #1945 | | Article #2074 | | Article #2118 | |
|  | Coef | Probabilistic (Benchmark Interval) | Coef | Probabilistic (Benchmark Interval) | Coef | Probabilistic (Benchmark Interval) | Coef | Probabilistic (Benchmark Interval) | Coef | Probabilistic (Benchmark Interval) |
| Percent Agreement | **1.00** | **(0.80 – 1.00)** | **1.00** | **(0.80 – 1.00)** | **1.00** | **(0.80 – 1.00)** | **0.75** | **(0.60 – 0.80)** | **1.00** | **(0.80 – 1.00)** |
| Gwet’s AC | **1.00** | **(0.80 – 1.00)** | **1.00** | **(0.80 – 1.00)** | **1.00** | **(0.80 – 1.00)** | **0.64** | **(0.60 – 0.80)** | **1.00** | **(0.80 – 1.00)** |

**Benchmark scale**

**<0.0000 Poor**

**0.0000-0.2000 Slight**

**0.2000-0.4000 Fair**

**0.4000-0.6000 Moderate**

**0.6000-0.8000 Substantial**

**0.8000-1.0000 Almost Perfect**

- 1. **Q45 – study type**

All articles reviewed were restricted to agreement on study type selected. Percent agreement of raters was perfect (1.0) across articles, except articles #927, #2074 and #2118 where agreement was 0.75. The chance-corrected Gwet’s AC among raters similarly showed perfect agreement among raters across articles, except the three articles listed above with 0.64 agreement.

|  | Article #423 | | Article #523 | | Article #788 | | Article #927 | | Article #1274 | |
| --- | --- | --- | --- | --- | --- | --- | --- | --- | --- | --- |
|  | Coef | Probabilistic (Benchmark Interval) | Coef | Probabilistic (Benchmark Interval) | Coef | Probabilistic (Benchmark Interval) | Coef | Probabilistic (Benchmark Interval) | Coef | Probabilistic (Benchmark Interval) |
| Percent Agreement | **1.00** | **(0.80 – 1.00)** | **1.00** | **(0.80 – 1.00)** | **1.00** | **(0.80 – 1.00)** | **0.75** | **(0.60 – 0.80)** | **1.00** | **(0.80 – 1.00)** |
| Gwet’s AC | **1.00** | **(0.80 – 1.00)** | **1.00** | **(0.80 – 1.00)** | **1.00** | **(0.80 – 1.00)** | **0.64** | **(0.60 – 0.80)** | **1.00** | **(0.80 – 1.00)** |
|  | | | | | | | | |  |  |
|  | Article #1645 | | Article #1829 | | Article #1945 | | Article #2074 | | Article #2118 | |
|  | Coef | Probabilistic (Benchmark Interval) | Coef | Probabilistic (Benchmark Interval) | Coef | Probabilistic (Benchmark Interval) | Coef | Probabilistic (Benchmark Interval) | Coef | Probabilistic (Benchmark Interval) |
| Percent Agreement | **1.00** | **(0.80 – 1.00)** | **1.00** | **(0.80 – 1.00)** | **1.00** | **(0.80 – 1.00)** | **0.75** | **(0.60 – 0.80)** | **0.75** | **(0.60 – 0.80)** |
| Gwet’s AC | **1.00** | **(0.80 – 1.00)** | **1.00** | **(0.80 – 1.00)** | **1.00** | **(0.80 – 1.00)** | **0.64** | **(0.60 – 0.80)** | **0.64** | **(0.60 – 0.80)** |

**Benchmark scale**

**<0.0000 Poor**

**0.0000-0.2000 Slight**

**0.2000-0.4000 Fair**

**0.4000-0.6000 Moderate**

**0.6000-0.8000 Substantial**

**0.8000-1.0000 Almost Perfect**

- 1. **Q52 – type of outcome**

All articles reviewed were restricted to agreement on type of outcomes selected. Percent agreement of raters ranged from substantial agreement (0.67) to perfect (1.0) across articles. The chance-corrected Gwet’s AC among raters ranged from moderate (0.47) to perfect agreement among raters across articles. This was expected since raters could select from a wide range of service, implementation, and impact outcomes.

|  | Article #423 | | Article #523 | | Article #788 | | Article #927 | | Article #1274 | |
| --- | --- | --- | --- | --- | --- | --- | --- | --- | --- | --- |
|  | Coef | Probabilistic (Benchmark Interval) | Coef | Probabilistic (Benchmark Interval) | Coef | Probabilistic (Benchmark Interval) | Coef | Probabilistic (Benchmark Interval) | Coef | Probabilistic (Benchmark Interval) |
| Percent Agreement | **1.00** | **(0.80 – 1.00)** | **1.00** | **(0.80 – 1.00)** | **0.67** | **(0.60 – 0.80)** | **1.00** | **(0.80 – 1.00)** | **0.75** | **(0.60 – 0.80)** |
| Gwet’s AC | **1.00** | **(0.80 – 1.00)** | **1.00** | **(0.80 – 1.00)** | **0.52** | **(0.40 – 0.60)** | **1.00** | **(0.80 – 1.00)** | **0.64** | **(0.60 – 0.80)** |
|  | | | | | | | | |  |  |
|  | Article #1645 | | Article #1829 | | Article #1945 | | Article #2074 | | Article #2118 | |
|  | Coef | Probabilistic (Benchmark Interval) | Coef | Probabilistic (Benchmark Interval) | Coef | Probabilistic (Benchmark Interval) | Coef | Probabilistic (Benchmark Interval) | Coef | Probabilistic (Benchmark Interval) |
| Percent Agreement | **0.58** | **(0.40 – 0.60)** | **0.67** | **(0.60 – 0.80)** | **1.00** | **(0.80 – 1.00)** | **0.75** | **(0.60 – 0.80)** | **0.75** | **(0.60 – 0.80)** |
| Gwet’s AC | **0.47** | **(0.40 – 0.60)** | **0.52** | **(0.40 – 0.60)** | **1.00** | **(0.80 – 1.00)** | **0.64** | **(0.60 – 0.80)** | **0.64** | **(0.60 – 0.80)** |

**Benchmark scale**

**<0.0000 Poor**

**0.0000-0.2000 Slight**

**0.2000-0.4000 Fair**

**0.4000-0.6000 Moderate**

**0.6000-0.8000 Substantial**

**0.8000-1.0000 Almost Perfect**

**Conclusion**

Across all articles reviewed, several inter-rater reliability statistics were generated. The overall percent agreement was the overall percent agreement was **86%, implying almost perfect agreement** among raters. The overall chance-corrected Gwet’s agreement coefficient was also 86%, indicating almost perfect agreement among raters. Agreement coefficients differed by articles, and by the implementation strategies, methods, study types and types of outcomes, depending on the covariate restrictions applied.

**References**

1. StataCorp. 2015. *Stata Statistical Software: Release 14*. College Station, TX: StataCorp LP.
2. Li Gwet, K. (2008). Computing inter-rater reliability and its variance in the presence of high agreement. *British Journal of Mathematical and Statistical Psychology*, (61), 29–48. https://doi.org/10.1348/000711006X126600
3. Gwet, K. L. (2015). *On Krippendorff’s Alpha Coefficient*. Retrieved from http://www.agreestat.com/research_papers/onkrippendorffalpha_rev10052015.pdf
4. Wongpakaran, N., Wongpakaran, T., Wedding, D., & Gwet, K. L. (2013). A comparison of Cohen’s Kappa and Gwet’s AC1 when calculating inter-rater reliability coefficients: a study conducted with personality disorder samples. *BMC Medical Research Methodology*, *13*(1), 61. https://doi.org/10.1186/1471-2288-13-61
